# Renal Artery Thrombosis Leading to Renal Infarct in a Patient With Recurrent Nephrolithiasis

**DOI:** 10.7759/cureus.38169

**Published:** 2023-04-26

**Authors:** Apurva Vedire, Steven Imburgio, Ritu Chakrabarti, Michael Levitt

**Affiliations:** 1 Internal Medicine, Jersey Shore University Medical Center, Neptune City, USA; 2 Hematology and Oncology, Jersey Shore University Medical Center, Neptune City, USA

**Keywords:** anticoagulation, flank pain, nephrolithiasis, idiopathic infarct, renal artery thrombosis, renal infarct

## Abstract

Renal infarction is a rare entity that presents similarly to other common renal conditions such as nephrolithiasis, which can often result in a missed or delayed diagnosis. As a result, a high degree of suspicion for this diagnosis is warranted in patients presenting with flank pain. We present a patient with recurrent nephrolithiasis who presented with flank pain. A subsequent workup revealed a renal infarct due to underlying renal artery thrombosis. We also explore if there was a possible mechanism between this event and his history of recurrent nephrolithiasis.

## Introduction

Renal infarction is a rare condition with an incidence of approximately 0.007% based on emergency department admissions [1]. Idiopathic renal infarct is a rarer phenomenon that can be seen in younger patients without typical cardiovascular risk factors [2]. Patients often present with flank pain, which can mislead physicians to suspect more common etiologies such as kidney stones or kidney infections. Without prompt diagnosis and treatment, patients risk prolonged ischemia that can result in permanent renal dysfunction [2]. 

## Case presentation

A 37-year-old male with a past medical history significant for recurrent nephrolithiasis presented to the emergency department with left lower quadrant and left-sided flank pain for two days duration. The pain was rated as 10/10 in severity, sharp in quality, non-radiating, worse with positional changes, and without alleviating factors. He reported associated diaphoresis and vomiting during this time due to the pain. The patient denied any fever, chills, dysuria, hematuria, chest pain, palpitations, or lower extremity swelling. He reported previous episodes of nephrolithiasis with confirmed stones in the left ureter and subsequently in the left ureterovesical junction that were treated with medical management. He denied recent prolonged immobilization, hormone therapy, known malignancy, recent travel, recent injury, or trauma to the flank area. He had no relevant past surgical history. He was a current smoker with a 17 pack-year smoking history, consumption of one alcoholic beverage per week, and denied any illicit drug use. There was no known personal or family history of venous thromboembolism. 

**Figure 1:**
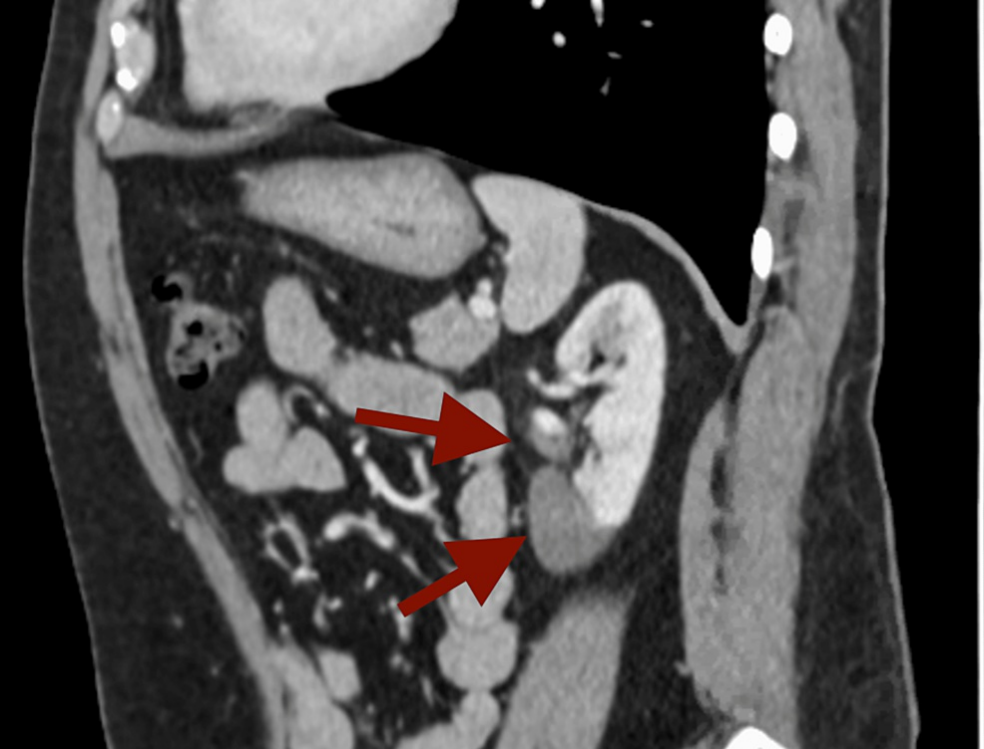
CT scan with contrast of the abdomen and pelvis depicting renal artery thrombosis with renal infarct in the mid to lower pole of the kidney

On admission, the patient was afebrile and hemodynamically stable. Physical examination was significant for left lower quadrant abdominal and left-sided costovertebral angle tenderness. The remainder of the exam was unremarkable. Laboratory findings showed leukocytosis with white cell count 11.9 10*3/uL (normal range 4.5-11 10*3/uL) and mild anemia with hemoglobin 13.1 g/dL (normal range 13.2-17.5 g/dL). There was no evidence of hepatic or renal dysfunction in laboratory studies. A CT scan with contrast of the abdomen and pelvis (Figure 1) showed moderate asymmetric patchy enhancement of the left mid to lower pole of the kidney, suspicious for renal infarcts. Additionally, there appeared to be nonopacification of the left lower pole renal artery, raising concern for thrombosis.

Hypercoagulable workup was negative for acquired causes of hypercoagulable state (Table 1). No mutations were identified in BCR-ABL1, P190 BCR ABL1, P210 BCR ABL1, and Janus kinase 2 (JAK2) V617F. After a discussion with hematology-oncology and vascular surgery specialists, it was decided that percutaneous thrombectomy or thrombolysis would not be beneficial in this case. The patient was immediately started on a heparin drip and later transitioned to oral anticoagulation.

**Table 1:** Laboratory results during hospital admission

Laboratory finding	Result	Reference range
Lupus anticoagulant	Not detected	Not detected
Anticardiolipin antibodies	<2 U/mL	<20 U/mL
Beta-2 glycoprotein antibody	<2 U/mL	<20 U/mL
Protein C activity	132%	70-180% normal
Protein S activity	73%	70-150% normal
Antithrombin III activity	94%	80-135% normal
Factor II activity	113%	70-150% normal
Factor V activity	106%	65-150% normal
Lactate dehydrogenase	329 U/L	91-200 U/L
Partial thromboplastin time	30 seconds	26-39 seconds
Prothrombin time	11.9 seconds	10.5-14.1 seconds
International normalized ratio	0.98	0.88-1.15

In the workup of this systemic thrombus, a comprehensive workup was conducted. An electrocardiogram (ECG) confirmed that the patient was in normal sinus rhythm. A transthoracic echocardiogram demonstrated normal left ventricular systolic function with no valvular vegetations. Bilateral lower extremity ultrasound was negative for deep venous thrombosis. Given the absence of any identifiable etiology, it was determined that the renal artery thrombosis and subsequent renal infarct were likely idiopathic in origin. He was discharged on oral anticoagulation with instructions for close outpatient follow-up. 

## Discussion

Renal infarction is often underdiagnosed due to the overlap in symptoms with more commonly occurring conditions like nephrolithiasis and pyelonephritis [3]. Often, unilateral flank pain is the classic presenting complaint [4]. Other symptoms can include nausea, vomiting, fever, and hypertension [5]. Elevated lactate dehydrogenase can be an important clue that can be checked on initial labs, along with hematuria and leukocytosis [3,4]. The workup for possible etiologies should include ECG, echocardiography, cardiac monitoring, and a thrombophilia panel [4]. The most sensitive and specific imaging modality available is renal artery angiography, however, this is an invasive test that is usually avoided [5]. A CT scan with contrast is one of the most commonly used imaging tests due to its non-invasive nature, easy availability, and ability to identify other conditions with similar presentations. Wedge-shaped areas of decreased attenuation signify acute segmental infarcts, whereas complete renal artery occlusion results in a “rim sign” where there is decreased attenuation throughout the renal parenchyma with the sparing of a rim of viable tissue in the capsule [5].

The most common etiology involves thromboembolic phenomenon in the setting of cardiac disease [3]. Other unusual causes include infective endocarditis, cocaine use, sickle cell, and connective tissue disorders [4]. The SARS-CoV2 (COVID-19) infection was also seen to cause renal infarcts possibly through mechanisms such as an increase in pro-inflammatory cytokines like tumor necrosis factor-alpha (TNF-a) and interleukin (IL)1, IL2, and IL6 [6]. Idiopathic renal infarcts should be considered in patients after other etiologies are ruled out such as risk factors for thromboembolism, disorders causing hypercoagulability, and renal vascular pathology [7]. Due to the rare incidence of idiopathic infarcts, the pathophysiology has not been well studied [7]. Spontaneous renal artery thrombosis is extremely rare [8]. The most common causes of such in-situ thrombosis include renal artery atherosclerosis and blunt abdominal trauma. Some case reports have shown rare causes such as polycythemia vera, renal transplant, pregnancy, renal surgery, and nephrotic syndrome [8]. 

The cornerstone of renal infarct treatment is the prompt restoration of renal blood flow prior to the development of partial or complete permanent renal injury [9]. The extent of infarction depends on the area of obstruction as well as available collaterals from other arteries. Systemic anticoagulation should be started once the diagnosis is confirmed to limit or prevent ischemic damage [5]. Due to the low incidence of patients preventing large-scale clinical trials, the optimal duration of anticoagulation is an area of controversy. However, patients found to have underlying prothrombotic conditions such as atrial fibrillation are usually continued on anticoagulation indefinitely [10]. Other treatment modalities that can be considered based on specific patient characteristics and institutional resources include thrombus aspiration, systemic intravenous thrombolysis, selective intra-arterial thrombolysis, open surgery, and revascularization with stents [9]. The most common long-term sequelae after renal infarction include renal dysfunction and persistent hypertension. Patients who receive prompt treatment have shown complete recovery of renal function with only a minority requiring dialysis [10].

Our patient had an extensive workup which was negative for common culprits of renal thrombosis and infarcts. Inflammation-induced thrombosis has complicated pathogenesis. Inflammation causes an increase in procoagulant factors and inhibits natural anticoagulant pathways. Chronic inflammation can even result in endothelial damage which causes the loss of physiologic anticoagulant and antiaggregant properties of the endothelium [11]. Studies have demonstrated that renal stones can cause inflammation, which can in turn lead to stone recurrence. In this way, a toxic cycle of kidney tissue injury and recurrent stone formation ensues [12]. Kidney stones can cause oxidative stress and trigger an inflammatory response with the release of pro-inflammatory cytokines like IL-1β and IL18 [13]. Pro-inflammatory cytokines such as interleukins play an important role in venous thromboembolism by promoting a procoagulant state [14]. Overall, more research is needed to help identify if there is a relationship between recurrent kidney stones and renal thrombosis. 

## Conclusions

Renal infarct should be considered in patients presenting with unilateral flank pain despite symptoms often occurring in other common conditions. A reasonable index of suspicion is warranted, even in the presence of previously known renal pathology such as nephrolithiasis, since a missed diagnosis may result in permanent renal impairment. Appropriate diagnostic workup with CT imaging should be considered in patients without typical prothrombotic risk factors, as renal infarcts due to renal artery thrombosis can also occur in young patients. Currently, there is a lack of information regarding the pathogenesis of idiopathic renal infarcts; recurrent nephrolithiasis may serve as a potential trigger for new-onset renal infarcts due to the pro-inflammatory state it induces. Overall, the prognosis of these patients appears to be positive with prompt diagnosis and initiation of anticoagulation. 
